# Knowledge-Guided “Community Network” Analysis Reveals the Functional Modules and Candidate Targets in Non-Small-Cell Lung Cancer

**DOI:** 10.3390/cells10020402

**Published:** 2021-02-16

**Authors:** Fan Wang, Shuqing Han, Ji Yang, Wenying Yan, Guang Hu

**Affiliations:** 1 Center for Systems Biology, Department of Bioinformatics, School of Biology and Basic Medical Sciences, Soochow University, Suzhou 215123, China; 20184212004@stu.suda.edu.cn (F.W.); 1730416005@stu.suda.edu.cn (S.H.); 20184212001@stu.suda.edu.cn (J.Y.); 2 State Key Laboratory of Radiation Medicine and Protection, Soochow University, Suzhou 215123, China

**Keywords:** non-small-cell lung cancer, protein-protein interactions, random walk with restart, functional modules, signaling transduction

## Abstract

Non-small-cell lung cancer (NSCLC) represents a heterogeneous group of malignancies that are the leading cause of cancer-related death worldwide. Although many NSCLC-related genes and pathways have been identified, there remains an urgent need to mechanistically understand how these genes and pathways drive NSCLC. Here, we propose a knowledge-guided and network-based integration method, called the node and edge Prioritization-based Community Analysis, to identify functional modules and their candidate targets in NSCLC. The protein–protein interaction network was prioritized by performing a random walk with restart algorithm based on NSCLC seed genes and the integrating edge weights, and then a “community network” was constructed by combining Girvan–Newman and Label Propagation algorithms. This systems biology analysis revealed that the *CCNB1*-mediated network in the largest community provides a modular biomarker, the second community serves as a drug regulatory module, and the two are connected by some contextual signaling motifs. Moreover, integrating structural information into the signaling network suggested novel protein–protein interactions with therapeutic significance, such as interactions between *GNG11* and *CXCR2*, *CXCL3*, and *PPBP*. This study provides new mechanistic insights into the landscape of cellular functions in the context of modular networks and will help in developing therapeutic targets for NSCLC.

## 1. Introduction

Cancer is a heterogeneous disease that actually refers to a collection of vastly different cellular states with dysregulated cell signaling and regulatory circuits [1]. Among them, lung cancers are the leading cause of cancer-related deaths worldwide [2]. The prognosis for lung cancer remains poor, although therapeutic developments including tyrosine kinase inhibitors and immunotherapy have promise [3]. Non-small-cell lung cancer (NSCLC) is the most common lung malignancy and is strongly related to gene aberrations and environmental influences [4,5]. Current genomic hallmarks for NSCLC include somatic mutations in PTPN11 (EGFR), SOS1 (KRAS), and STK11 (neutrophil degranulation) [6], and future therapeutic decisions will be helped by an increased understanding of other NSCLC-related pathways, such as EGFR, PI3K/AKT/mTOR, RAS/MAPK, and JAK/STAT [7]. Currently, there are efforts not only to elucidate the mutational and gene expression data, but also to present the emerging proteomic landscape of NSCLC [8], which provides a resource for the comprehensive elucidation of aberrant biological process, candidate biomarkers, and therapeutic targets. An integrative proteomic analysis suggested some prognosis-associated proteins and pathways in early stage NSCLC [9], while multi-omics clustering has also revealed that EGFR, KRAS, and STK11 are candidate drug targets for NSCLC [10]. Although recent omics studies of NSCLC have advanced our understanding of tumor biology and accelerated targeted therapy, the complex landscape of NSCLC, particularly for cellular communications, remains largely open.

Most cellular signaling and surveillance circuits are physically maintained through a dense network of protein–protein interactions (PPIs) [11,12]. Therefore, biological networks are promising when trying to uncover the causes of complex diseases [13] such as cancers and will help in the next phases of drug design [14]. This new paradigm reflects the fact that human diseases are not caused by single molecular defects but are driven by complex interactions among a variety of molecular mediators [15]. The network-based methods have developed a plethora of topological parameters for discovering biomarkers [16], disease-associated genes [17], and drug targets [18,19]. Hub genes with higher node degree in PPI networks have been predicted to be diagnostic biomarkers for NSCLC and some have been experimentally validated, such as *NCAPH* [4]. In addition to node prioritization, edge-based topological parameters, such as shortest path, mutual neighbors, between and cross communities, were also used to rank pair-wise interactions in cancer-related PPI networks [20,21].

Network biology provides a quantitative tool to elucidate the structural and functional architecture of the hidden higher-level organization of cellular communication. The community analysis of PPI networks is a process, in which networks are divided into several topological modules. Proteins within the same community may serve as interacting molecular machines, driving a common biological process [22]. PPI networks-based community analysis allows us to ponder the heterogeneity of cancer [23], and reduces the complexity of disease networks. Currently, several community analysis methods, such as ClusterONE [24], ModuLand [25], Molecular Complex Detection (MCODE) [26], MTGO [27], and PS-MCL [28], have been successfully proposed to identify target proteins and drug repurposing [29]. Combined with community analysis performed by MCODE, *CCNB1* has been predicted as a hub gene in a particular module of NSCLC [30], while *FOXM1* and *MYBL2* are predicted to be “Key Regulators of Cell Proliferation” in NSCLC [31]. Additionally, more key modules and genes in NSCLC have been identified by using co-expressed modules and hub gene analysis from Weighted Gene Co-Expression Network Analysis and PPI network analysis, respectively [32]. However, no matter what criterion is used, different community identification approaches can reveal different functional modules. A recent assessment revealed that top-performing community identification algorithms could recover complementary trait-associated modules [33].

If the end goal is drug discovery, a systems-level approach including the identification of key nodes, edges, and communities is not enough. The wealth of structural knowledge in PPI networks can help to partially address this goal [34]. Therefore, by mapping NSCLC-associated mutations on the interface regions of protein interactions may provide structural and dynamical evidence for understanding cellar pathway transformation and the genotype–phenotype relationship [35]. By mapping genomic profiles of driver gene mutations onto the structure of epidermal growth factor receptor (EGFR), four druggable mutations have been discovered that can be used to design personalized NSCLC treatments [36]. Based on structural-based PPI networks [37], dynamics information can be integrated to generate mutational hotspot communities, which significantly increases the sensitivity of cancer driver genes [38]. Armed with these structural insights, the protein binding poses and affinities bring breakthroughs toward understanding the molecular basis of cell-cell communication [39].

**Figure 1:**
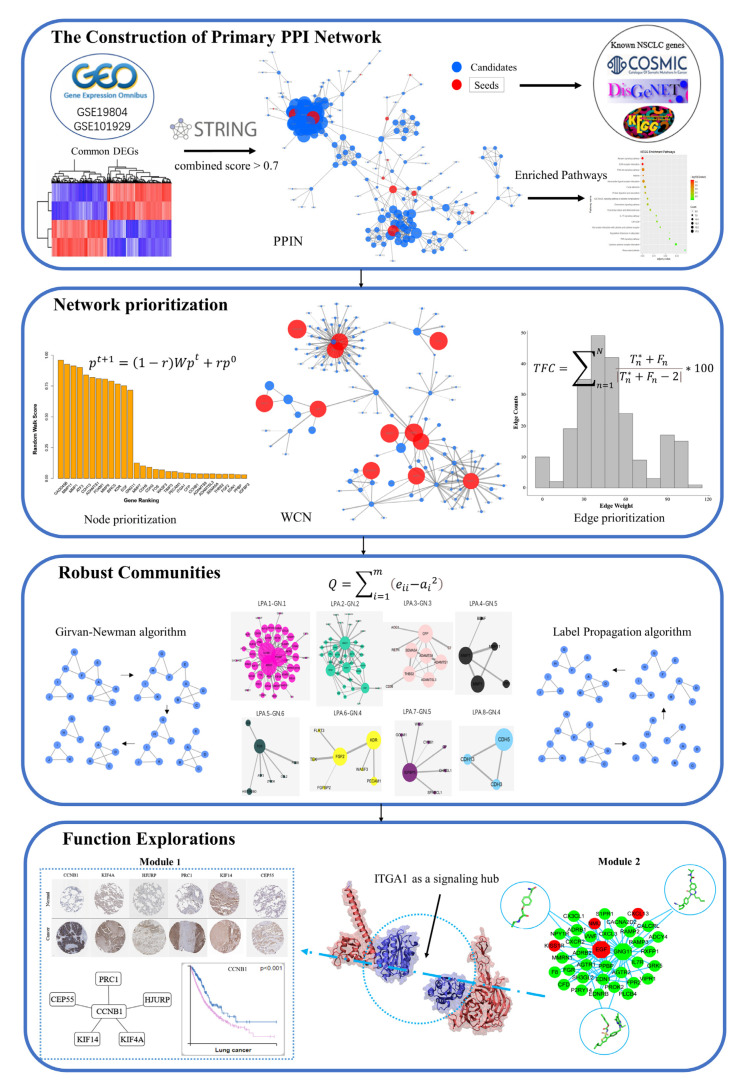
The ne-PCA workflow, which contains four major parts: the construction of primary PPI network, the prioritization of nodes and edges, the identification of robust communities, as well as the function explorations of the “community network”.

In this study, we developed a knowledge-guided and network-based methodology to understand the complex molecular mechanism among different NSCLC functional modules; the complete methodology of this study is presented in Figure 1. First, the random walk with restart (RWR) algorithm was used to rank and predict key genes based on seed genes. Additionally, a new score was defined for edge prioritization to construct the weighted core network that combined topological features and biological similarity of edges. Then, the Girvan–Newman (GN) algorithm and Label Propagation analysis (LPA) were combined to generate “community networks”, which are molecular networks connected by different functional modules. Along the pipeline, the functional significance of different modules and key genes were further verified by published experimental data and structural modeling. Accordingly, we hope that the detected “community networks” could define the inner working of the cellular processes in lung cancer and highlight potential therapeutic vulnerabilities of NSCLC and other complex diseases.

## 2. Materials and Methods

### 2.1. Data Sets

Two independent NSCLC expression microarray datasets based on the same platform (GSE19804 [40] and GSE101929 [41]) were obtained from the NCBI Gene Expression Omnibus database (Table 1). In total, the datasets contained 186 samples, including 92 NSCLC and 94 normal samples. Expression values of the predicted genes were selected from The Cancer Genome Atlas (TCGA) [42], including 126 samples, with 67 NSCLC and 59 normal samples. The NSCLC-related genes that were used as seed genes (Table 2) were collected from three authoritative databases, including Kyoto Encyclopedia of Genes and Genomes (KEGG) [43], Cancer Gene Census (CGC) [44] and DisGenet [45]. Limma package [46] in R/Bioconductor software was applied to identify the differentially expressed genes (DEGs) with *p*-values adjusted by the Benjamini–Hochberg method. Only genes with adjusted *p*-values < 0.001 and |FC| >2 were chosen as DEGs.

### 2.2. Network-Based Methodology

At the preprocessing stage, a PPI network of DEGs was constructed according to the STRING database [47] with the score over 0.7 and all active interaction sources except text mining. Then, seed genes associated with NSCLC were mapped onto the PPI network to construct the seed-based PPI network. Given this network structure, we have developed a novel algorithmic framework, termed node and edge Prioritization-based Community Analysis (ne-PCA) to generate “community networks” in NSCLC. This process includes three major steps: (1) node prioritization; (2) edge prioritization; and (3) community identification. The code for our network-based algorithm can be found at Github repository (https://github.com/CSB-SUDA/ne-PCA).

**Step 1: Node prioritization.** In this step, the RWR algorithm [48,49], which is a classic ranking algorithm, was used for node prioritization; thus identifying key genes by evaluating the proximity from seed genes in the primary PPI network. Starting from seed genes, an iterative walker transitions from its current node to a randomly selected neighbor starting at a given source node with restart of the walk at every time step at node *s* with probability *r*. The associated iteration equation is defined as:

(1) $$p^{t+1}=\left(1-r\right)Wp^{t}+rp^{0}$$

where *W* is the column-normalized adjacency matrix of all nodes in the network; *r* is the restart probability; $p^{0}$ is the initial weight vector based on a certain seed, *A*; and $p^{t}$ are the vectors in which *a*th walking holds the probability of being at node *a* at time *t*. Through multiple iterations, $p^{t}$ will approach a certain probability distribution, where $p^{t+1}$ is approximately equal to $p^{t}$. As such, the RWR algorithm calculates the similarity or closeness between seed gene *i* and each other node *j*, based on Equation (1), whereby all possible paths between the two genes within the PPI network are taken into account. RWR was performed under 0.8 restart probability via R package dnet [50], and $p^{t}$ was the RWR score used for ranking nodes.

**Step 2: Edge prioritization.** Edge betweenness and Gene Ontology (GO, R package GOSemSim version 2.8.0) semantic similarity analysis are two adapted parameters for edge prioritizations that are used to evaluate the topological and biological importance of edges. Edge betweenness [51] defines the number of shortest paths between all possible pairs of vertices in a graph that pass through the edge. High edge betweenness is also associated with pairs of residues that are important for communication within the PPI network. To be more compatible with the conduction of functional signals in real biological system, that is, the possibility of information spreading through a certain interaction, the biological functions of the interactors need to be considered. It is generally believed that if two interacting gene products (proteins) have more similar function such as more GO annotations, then their interactions have higher confidence. Thus, GO semantic similarity can be used as the weight of edges in PPI network, which is more biological meaning [20]. In our work, the ‘Wang method’ [52] was used to study functional similarity as it determines the semantic similarity of two GO terms based on both the locations of these terms in the GO graph and their relations with their ancestor terms:

(2) $$S_{A}\left(t\right)=\{\begin{array}{c} 1,\;t=A\\ max\{w_{e}\times S_{A}\left(t^{\prime }\right)|t^{\prime }\in \mathrm{children}\;\mathrm{of}\;\left(t\right)\},\;t\neq A \end{array}$$

where *w_e_* is the semantic contribution factor for edge e∈EA linking term *t* with its child term *t*′. Term *A* contributes to itself and is defined as 1. After obtaining the *S*-values related to term *A*, the semantic value of GO term *A*, SV(A), was calculated as:

(3) $$SV\left(A\right)=\sum_{t\in T_{A}}S_{A}\left(t\right)$$

Thus, given two GO terms *A* and *B*, the semantic similarity between them is defined as:

(4) $$sim\left(A,B\right)=\frac{\sum_{t\in T_{A\cap }T_{B}\;}S_{A}\left(t\right)+S_{B}\left(t\right)}{SV\left(A\right)+SV\left(B\right)}$$

where $S_{A}\left(t\right)$ is the *S*-value of GO term *t* related to term *A*, and $S_{B}\left(t\right)$ is the *S*-value of GO term *t* related to term *B*.

Based on the ability and probability weight of information dissemination through PPIs, a new score named Topological-Functional Connection (TFC) was proposed for ranking PPIs. Mathematically, TFC is defined as

(5) $$TFC=\sum_{n=1}^{N}\frac{T_{n}^{*}+F_{n}}{\left|T_{n}^{*}+F_{n}-2\right|}*100$$

(6) $$T_{n}^{*}=\frac{T_{n}-Min_{T}}{Max_{T}-Min_{T}}$$

where *N* represents the number of interactions, and $T_{n}$ and $F_{n}$ represent edge betweenness and GO semantic similarity of interaction *n*. As such, the TFC score can be used to identify key protein interactions by integrating network topology and biological characteristics, which supplement missing functions in traditional network information flow.

**Step 3: Identifying network communities.** An integration method for the identification of network modules was also proposed. First, the weighted core network (WCN) was extracted from the PPI network according to the seed-based random walk score. In this WCN, only the top 10% of scored genes and seeds with their neighborhoods were chosen as nodes, which were connected by the edges weighted by TFC scores. Then, two common cluster methods were used to detect communities of WCN. To determine the inherent module attributes in the core net, we use the weighted GN algorithm [53] to achieve a top to down module discovery, which is the most classic community discovery algorithm based on the use of the edge betweenness as the partitioning criterion. The GN algorithm is a split-level hierarchical clustering algorithm, and the module was identified by continuously deleting edges in the network. LPA was also performed to achieve down to top module discovery by using the information of the prior seed genes [54]. An initial label was given to the seed gene in advance, and the gene with the largest labeling of neighboring nodes was used as its label in each iteration. The TFC score was set as the weight of the edges and the modularity *Q* could be optimized automatically. Modularity *Q* is the quality function of the network division:

(7) $$Q=\sum_{i=1}^{m}\left(e_{ii}-a_{i}{}^{2}\right)$$

where $e_{ii}$ is the fraction of edges between modules *i* and *j*, and *a_i_* is the fraction of edges connected to the nodes in module *i*. This modular structure is found by maximizing the modularity in an iterative manner. All nodes in the network were assigned to independent modules in the beginning, and the algorithm progressively merged two communities that best increased the modularity of the resulting network. Merging nodes and modules continued until there was no further increase in the modularity of the network. Lastly, a hypergeometric test was performed for each pair of modules to integrate the similarity part in different model results. The common parts of modules with significant *p*-values (*p* < 0.01) were screened out as robust modules.

### 2.3. Functional and Pathway Enrichment Analyses

R package clusterProfiler [55] was used for GO and KEGG pathway enrichment analysis. Terms with corrected *p* value < 0.05 were selected as significantly enriched terms.

### 2.4. Performance of Candidate Biomarkers and Validating Predicted Genes

To evaluate the performance of the predicted genes as prognostic biomarkers, Kaplan–Meier analyses with log-rank tests were performed for 994 TCGA NSCLC samples including patients’ clinical information and RNA expression from the pathology atlas in *Human Protein Atlas* [56]. The best expression cut off for survival analysis in *Human Protein Atlas* was used for sample grouping.

### 2.5. Permutation Test for Community Network and Comparison with Other Methods

Permutation test for final community network from ne-PCA was performed according to significance in module score W based on Markov random field (MRF). The detail of this method can be found in the recent work [57]. The module score W of network M was defined as:

(8) $$W\left(M\right)=\;\frac{1}{\sqrt{m}}\sum_{i\in C_{1}}f_{i}-\frac{1}{k}\sum_{u,v\in C_{2}}{\left(\frac{f_{u}}{\sqrt{d_{u}}}-\frac{f_{v}}{\sqrt{d_{v}}}\right)}^{2}MI\left(u,v\right)$$

where m is the number of nodes in *M*, *k* is the number of interactions in *M*, *C*_1_ and *C*_2_ are the set of seed genes and non-seed genes in *M*, *f_u_* and *f_v_* are expression differences (negative logarithm of p value) assessed by t-test between tumor and normal samples from GSE101929, *d_u_* and *d_v_* are the degree of non-seed genes *u* and *v* in primary PPI network, and MI(u,v) is the mutual information of non-seed genes *u* and *v* from expression profile, respectively. In our work, we performed 10,000 random experiments with the same number of samples as the community network under test. Scores significantly greater than the random ones (*p* < 0.05) were considered significant.

In addition, some commonly used network-based methods were also performed for the PPI network analysis to compare with ne-PCA. Degree, betweenness, closeness, and clustering coefficient were calculated for node prioritization [58]; edge betweenness for edge prioritization [20]; and four network clustered methods including ClusterOne [24], Moduland [25], MCODE [26], and MCL [28], for community analysis.

### 2.6. Constructing the Target-Drug Network

The drug targets in Module 2 and corresponding drugs screened from Drugbank [59] and Therapeutic Target Database [60] were used as nodes. Their interactions were used to construct a target-drug network. The network was constructed and visualized using Cytoscape [61].

### 2.7. Structural Modeling of PPIs

The structural modeling of sub-networks and PPIs was performed by PRISM [62,63], which is a powerful template-based algorithm that has prior interface knowledge of known 3D structures of PPI complexes to predict structural interactions of target proteins. If the experimental 3D structure of the target protein was missing from the PDB, we built models of that protein by exploiting the I-TASSER server [64]. For the modeled protein complex, binding energies were calculated using FoldX [65] to measure stability. Druggabilities of PPIs were evaluated by druggability scores (DS) calculated by Fpocket [66]. The score chosen was the highest score of each structure and classified as: 0.0–0.5: non-druggable; 0.5–0.7: druggable; and 0.7–1.0: highly druggable.

## 3. Results

### 3.1. Knowledge-Guided Construction of a WCN Based on Seed Genes

**Figure 2:**
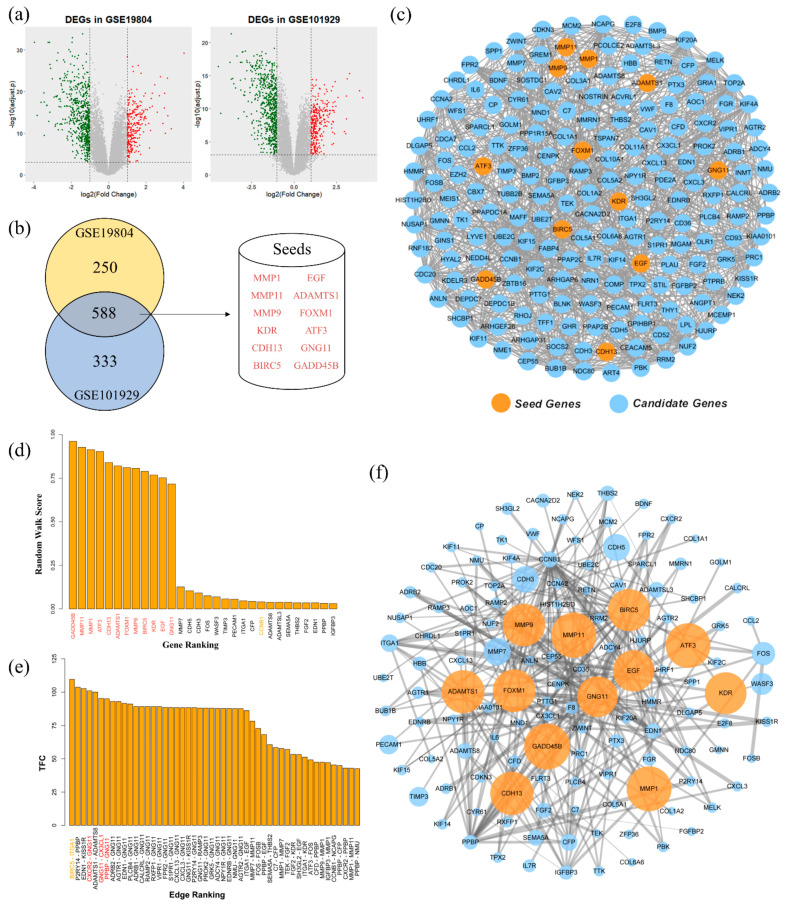
The construction of Weighted Core Network (WCN). (**a**) Volcano plot of DEGs in two datasets of NSCLC. The horizontal line at false discovery rate (FDR) = 0.001; vertical line at |log2FC| = 1. (**b**) Venn diagram shows the overlap of DEGs in two datasets, while the numbers of overlapping DEGs include both up- and down-regulated genes. Twelve NSCLC-related genes found in the overlap DEG sets are also listed. (**c**) The primary seed-based NSCLC PPI network, in which orange nodes represent seeds and blue nodes, represent candidates. (**d**) Network node prioritization by the RWR scores. (**e**) Network edge prioritization by the TFC scores, whose values are defined based on edge betweenness and GO semantic similarity. (**f**) The topology of WCN, in which the seeds were represented by orange nodes. Node sizes are denoted by RWR scores and edge thickness by TFC scores.

Statistical analysis of NSCLC and adjacent normal lung tissue samples identified DEGs that were significantly abnormally expressed in tumor tissues. In total, 258 up-regulated and 580 down-regulated DEGs were identified from GSE19804, and 295 up-regulated and 626 down-regulated DEGs were identified from GSE101929. Figure 2a shows volcano plots reflect the distribution of DEGs according to Fold Change and FDR. The Venn diagram of 588 overlapping DEGs between the two GEO datasets were found in Figure 2b and include 155 up-regulated and 433 down-regulated DEGs. Additionally, 12 known NSCLC-related genes were found among the overlapping DEGs from three curated databases of KEGG, CGC, and DisGenet, including *MMP1*, *MMP9*, *MMP11*, *KDR*, *CDH13*, *BIRC5*, *EGF*, *ADAMTS1*, *FOXM1*, *ATF3*, *GNG11*, and *GADD45B*. After applying the interaction score and source filter in the STRING database, the primary PPI network was constructed by overlapping DEGs. Thus, 12 NSCLC-related genes were defined as seeds and mapped into the primary PPI network to construct the seed-based PPI networks. Accordingly, the NSCLC PPI network that contains 190 nodes and 1128 edges was obtained (Figure 2c), while 12 NSCLC-related seed genes are highlighted in orange. The distribution of degree of such PPI network is shown in Figure S1a. KEGG pathway enrichment analysis indicated that the seed-based PPI network involved tumor-related signal transduction pathways, such as extracellular matrix (ECM) receptor interaction, PI3K/Akt signaling, TNF signaling, as well as some basic biological processes, such as protein digestion and absorption, cell cycle progression, and cytokine–cytokine receptor interaction (Figure S2).

Nodes of PPI network were prioritized by performing RWR, which used the knowledge from collected seed genes. Some genes that were highly related to seeds were identified according to the node prioritization (Figure 2d). In particular, the top 10 ranked genes were *MMP7*, *CDH5*, *CDH3*, *FOS*, *WASF3*, *TIMP3*, *PECAM1*, *ITGA1*, *CFP*, *CCNB1*, *ADAMTS8*, *ADAMTSL3*, *SEMA5A*, *THBS2*, *FGF2*, *EDN1*, *PPBP*, and *IGFBP3*. Then, the un-weighted core network for NSCLC was constructed by extracting the 12 seed genes and the top 10% genes from node prioritization. For comparison, some commonly used topological metrics including degree, closeness, betweenness, and clustering coefficient of the whole PPI network were calculated, and the genes ranked by each parameter are shown in Table S1. By investigating the topological parameters of seed genes, their distributions show that their biological importance cannot been predicted by their top ranked values. As shown in the scatter plot (Figure S3), only *BIRC5* shows large values for all topological parameters.

Additionally, a new score named TFC was defined as an edge parameter and was obtained by integrating edge betweenness and GO semantic similarity of interactions. As such, by mapping TFC onto each interaction in the core network, a knowledge-guided WCN was constructed (Figure 2f). The WCN contains 130 nodes and 245 edges, while the distribution of degree shows that it retains the scale-free property of primary PPI network (Figure S1b). Additionally, the final WCN not only considered network topology, but also contained biological information of gene function. Edges in the WCN were also prioritized by the newly defined TFC score, and important interactions have been predicted. The distribution of TFC scores is relatively even, while the *BIRC5*–*ITGA1* and *PPBP*–*P2RY14* interactions represent the two edges with highest TFC values (Figure 2e). By only using edge betweeness, some high ranked PPIs can also be predicted, for example, *BIRC5*-*ITGA1* has the highest edge betweeness. The comparison results between edge betweeness and TFC are shown in Figure S4. Although some topological important interactions can be predicted by edge betweeness (Figure S4a), the potential biological important interactions consisting of G Protein Subunit Gamma 11 (*GNG11*) cannot be captured, which are all ranked in the top list of TFC (Figure S4b). This interesting finding suggests a key role of *GNG11*, as it may be involved in key interactions that need further investigation.

### 3.2. Defining the Community Network for the NSCLC

**Figure 3:**
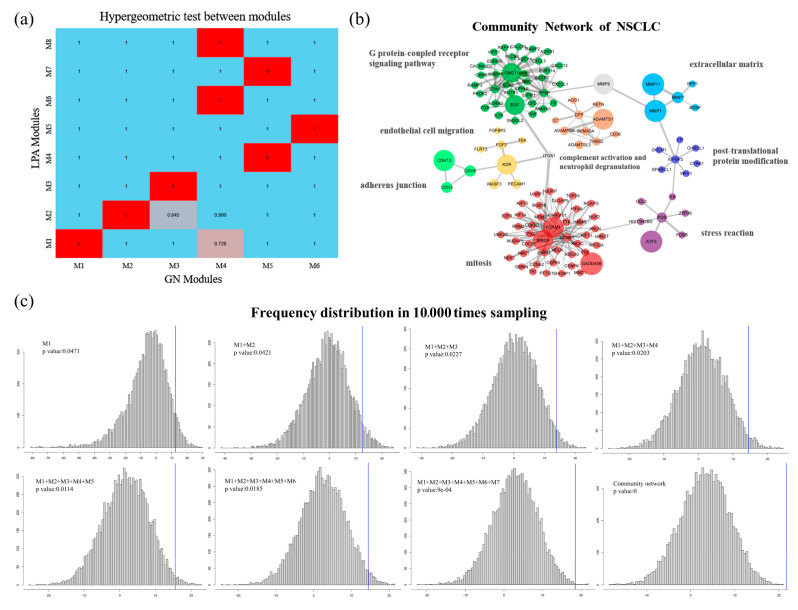
Community analysis of WCN. (**a**) Significance of the correlation test between each pair of communities. (**b**) The community network based on robust communities, in which communities were mapped by different colors and labeled by their GO biological process. (**c**) Module score distribution of 10,000 random cumulative experiments under MRF, and results from only including module 1 to all modules in “community network” are shown. The *x* axis and *y* axis, respectively, represent the module score and frequency of the sample in this score. The blue vertical line is the position of our network score.

In this section, a global community identification was conducted for the WCN, and six communities were found with the GN model and eight communities were detected with the LPA model (Figure S5). Their divisions were on the same level, while the modularity *Q* of GN and LPA were 0.65 and 0.62, respectively. The communities tended to be consistent overall across two models, however, there were slight differences in some aspects, indicating that these regions were not totally robust. The cluster of *ITGA1* connected with collagen genes (*COL1A1*, *COL1A2*, *COL5A1*, *COL5A2*, *COL6A6*) belonged to the largest community in LPA, while it was classified into an individual community in GN. There were also some specific modules detected exclusively by different methods, such as *HBB/MMP9/PTX3/TIMP3* in GN, and *CDH3/CDH5/CDH13* and *IGFBP3/WFS1/CHRD-L1/CP/CYR61/GOLM1/SPARCL1* in LPA. A hypergeometric test of each pair of communities from the two models was further performed to determine robust communities with high correlation (Figure 3a). Ultimately, the WCN was partitioned into eight robust communities, including two large communities and six small communities, forming a “community network” (Figure 3b). According to the size (node numbers) of the communities, we called the top two communities as module 1 (M1, red community) and module 2 (M2, green community). The community network revealed a functional map of the cell in which genes of similar biological processes clustered in each community. For example, GO enrichment analysis showed that the biological process of the modules 1 and 2 corresponded to “mitosis” and “G protein-coupled receptor signaling pathway”, respectively.

Within the community network framework, there are several small communities whose deletion will destroy the information transmission of the entire network. These are defined as connected motifs. The violet community (M4) was centered on the proto-oncogene c-Fos (*FOS*), which is a regulator of cell proliferation, differentiation, and transformation, and was related to “stress reaction”. The blue motif (M6) centered with Insulin Like Growth Factor Binding Protein (*IGFBP3*), primarily involves “post-translational protein modification”. The light blue motif (M7) contains the Matrix Metallopeptidase (*MMP*) protein family, which is involved in breaking down extracellular matrix in normal physiological processes. Additionally, the three branch communities included (M3) A disintegrin and metalloproteinase with thrombospondin motif (*ADAMTS*) protein family, (M5) vascular endothelial growth factor receptor (*KDR*), and (M8) the cadherin superfamily, which were clustered together, and corresponded to the biological processes “complement activation and neutrophil degranulation”, “endothelial cell migration”, and “adherens junction”, respectively.

To investigate the performance of our cluster method, large-scale random test based on MRF was carried out. The main result of the test from cumulative experiments showed that the community network of NSCLC was significant in module score W (*p* < 0.05, Figure 3c), which means that the community network was independent from random network under MRF. We specifically make use of seed genes to generate the final community network, a specific sub-network of NSCLC compared to the random division (normal distribution) on the primary network. Additionally, our results were also compared with four other state-of-the-art module detection algorithms (Figure S6 and Table S2). Overall, the core parts of these modules among these methods are consistent. We compare our results with them practically by focusing on modules 1 and 2. Although module 1 in MCL and ClusterOne include more nodes with smaller RWR score, some seed genes cannot be detected by other algorithms, such as *GADD45B* and *EGF*. For module 2, ne-PCA can detect more genes than it can in MCODE, moduland, and ClusterOne. We suppose the reason is that other algorithms are based only on topological properties, and then some biological similar genes cannot be clustered with low topological similarity.

**Figure 4:**
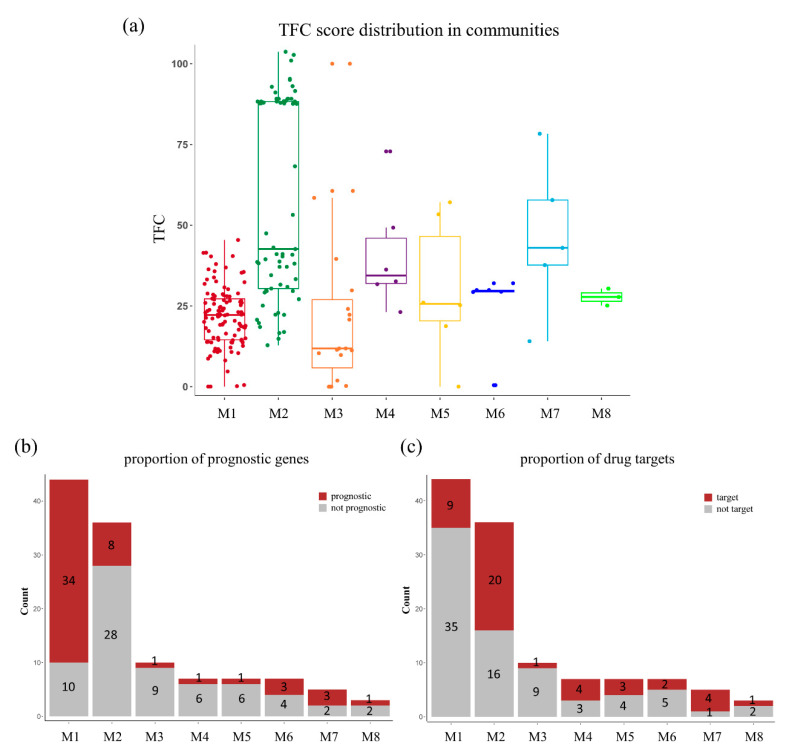
Topological and functional diversity of the community network. (**a**) Weights of edges in different communities. Distributions of (**b**) prognostic genes and (**c**) known drug targets in each module.

To primarily investigate the topological and functional diversity of these modules and motifs, we further evaluated the distributions of edge weights, prognostic genes, and drug targets among communities. As shown in Figure 4a, module 2 and the *MMP* motif (M7) showed higher TFC scores than other communities, which meant that those communities contained denser edges that may contribute to greater signal transmission in the topological view. In comparison with other small motifs, module 1 and 2 are the two largest communities that consist of genes with similar biological functions, which are of extreme importance. For their functional diversities (Figure 4b), module 1 contains the most prognostic genes (34/44), while module 2 (Figure 4c) includes the most drug targets *(26/36)*. Therefore, we suggested that these two modules have different biological implications, i.e., module 1 is a disease-related module and module 2 belongs to a drug target module. The community network not only divided the PPI network into individual functional modules, but could also decipher complex regulatory relations from the global network level. The fact that the *MMP* motif not only has the highest average TFC scores, but also consists of the highest percentage of drug targets, suggests its regulatory role. In our community network, *MMP9* serves as a bottleneck that connects module 1 and the *MMP* motif. Accordingly, our community network together with the skeleton, bottlenecks, and bridges, allowed us to define a module space for performing biological functions. As such, the biological roles of module 1 and module 2 will be explored in detail in the following sections.

### 3.3. Module 1 Represents a Significant Diagnostic Module Biomarker

**Figure 5:**
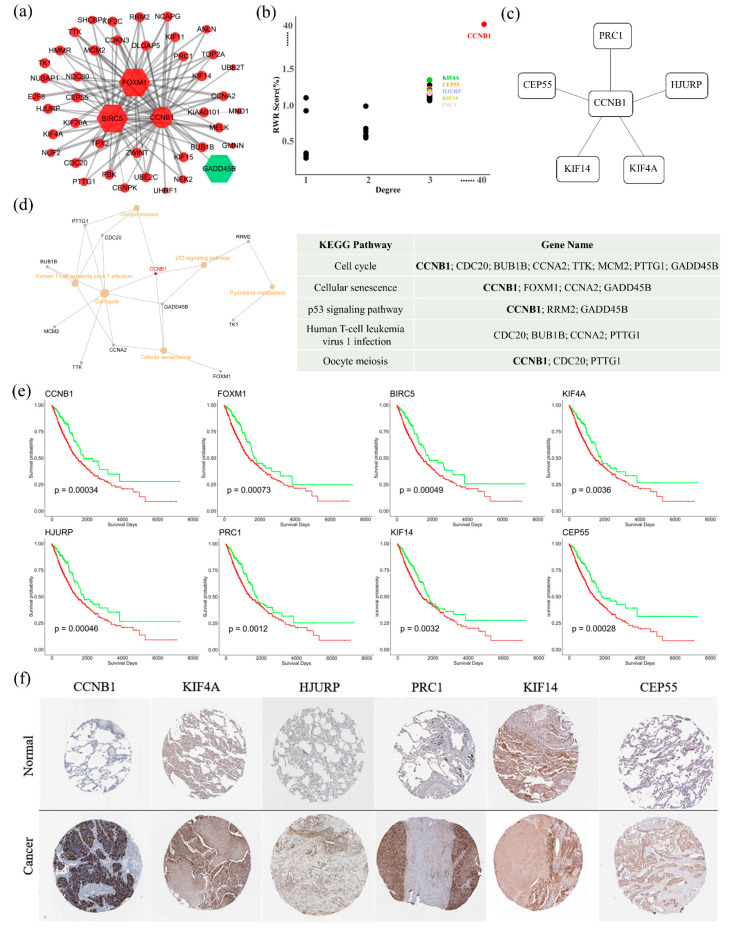
Clinical function of module 1. (**a**) The topological representation of Module 1. (**b**) A scatterplot showing the distribution of degree and RWR score. (**c**) *CCNB1*-centered sub-module. (**d**) Pathway enrichment analysis for genes in module 1, sorting by FDR in ascending order, while *CCNB1* was involved in all top three pathways. (**e**) Survival analysis for genes in *CCNB1*-centered sub-module. Red lines represent sample groups with high gene expression, while green lines represent sample groups with low gene expression. (**f**) The protein level expression of the genes in *CCNB1*-centered sub-module was higher in NSCLC than in normal cells, which were obtained from Human Protein Atlas (https://www.proteinatlas.org/, January 2021).

Module 1 is the largest robust community, containing 44 nodes, 3 seed genes (*BIRC5*, *FOXM1*, and *GADD45B*), and 106 interactions (Figure 5a). Interestingly, genes of the largest community were almost all up-regulated in NSCLC except for the seed gene *GADD45B*. Up-regulated overall expression of the largest community may be related to tumor biology at the systems level. To gain topological insights into module 1, we examined both RWR score and degree for each node (Figure 5b). The comparison showed that *CCNB1* had both the highest degree and RWR score among the predicted genes. As a hub gene, *CCNB1* connects with many other genes, among which *CEP55*, *PCR1*, *HJURP*, *KIG14*, and *KIF4A* had the highest RWR scores and relatively high degrees. This suggests that *CCNB1* and these five connected genes comprise a critical sub-network for module 1 (Figure 5c). Additionally, pathway enrichment analysis showed that module 1 was enriched in six KEGG pathways (FDR < 0.05, Figure 5d and Figure S7). The top three pathways were response to cell cycle, cellular senescence, and p53 signaling pathway, which are basic biological processes that participate in various disease mechanisms, especially the occurrence and development of tumors. Thus, the key role of *CCNB1* in this NSCLC-related module was affirmed, as it is involved in all of these pathways.

Then, we conducted a prognostic analysis using TCGA data. Kaplan–Meier analysis with the log-rank test was performed on 994 NSCLC samples, including patients’ clinical information and RNA expression from the pathology atlas in *Human Protein Atlas.* The best expression cut off for survival analysis in *Human Protein Atlas* was used for sample grouping. As described above, most of the genes in module 1 were prognostic, with high expression unfavorable in NSCLC (*p* < 0.01). Survival curves of the genes in the *CCNB1*-centered sub-module are shown in Figure 5e. High expression of the six genes was associated with poor survival. The remaining curves are available in Figure S8. In total, 16 genes had extreme prognostic significance (*p* < 0.001): *BIRC5*, *FOXM1*, *CEP55*, *NEK2*, *CDKN3*, *HMMR*, *TOP2A*, *TK1*, *HJURP*, *ANLN*, *CCNA2*, *TPX2*, *MELK*, *KIF11*, and *CCNB1*. Moreover, based on the immunohistochemical staining results in Human Protein Atlas, protein levels of genes in the *CCNB1*-centered sub-module were consistent with their mRNA expression, i.e., their protein levels were also higher in NSCLC compared with normal samples (Figure 5f).

### 3.4. Module 2 Suggests Potential Drug Targets for NSCLC Treatment

**Figure 6:**
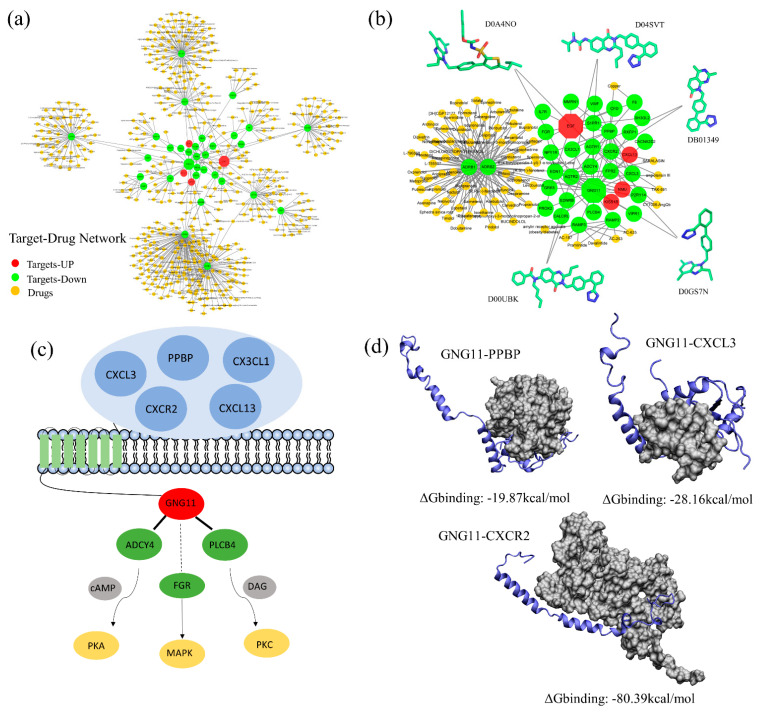
Functional exploration of module 2. (**a**) The target-drug network based on PPI of module 2, while red and green nodes stand for proteins, and yellow nodes denote drugs. (**b**) Note that *ADRB1*-*ADRB2* share many drugs, while *AGTR1*-*AGTR2*, *RAMP2*-*RAMP3*, and the edge *F8*-*PPBP* share a common drug. (**c**) The simple schematic diagram of chemokine signaling pathway. (**d**) Structural models for *GNG11*–*PPBP*, *GNG11*–*CXCL3*, and *GNG11*–*CXCR2* interactions. Atoms of interface residues are represented with balls.

The second most robust community was module 2, which contained 36 nodes, three seed genes (*EGF*, *NMU*, and *KISS1R*), and 65 interactions (Figure 6a). In contrast to the largest community, the second module was mostly a community of down-regulated genes, which means that their expression was significantly decreased in NSCLC tissues compared with normal tissues. To comprehensively elucidate the mechanism of this community, three levels of analysis were performed. By screening out the Drugbank and TDD databases, we found that most nodes of this community corresponded to known drug targets (Table S3), including two seed genes (*EGF* and *KISS1R*). The target-drug network contained 26 targets and 479 drugs. Among them, the target pair *ADBR1* and *ADBR2* share 63 drugs. Other examples include *AGTR1*-*AGTR2*, *RAMP2*-*RAMP3*, and *F8*-*PPBP*, which share some common drugs (Figure 6b). Additionally, *F8*-*PPBP* comprises an edge with high a TFC score in module 2. In fact, interactions in the second module had the highest TFC scores among other communities, while two interactions between know drug targets (*P2RY14*-*PPBP* and *EDN1*-*KISS1R*) were ranked as the top two highest TFC scores. As such, most genes that consist of module 2 are known drug targets, highlighting the importance of these core modules as therapeutic opportunities.

Next, pathway enrichment analysis showed that module 2 performed its biological functions mainly through various signal transduction pathways (Figure S5). Among them, the two most significant pathways were neuroactive ligand–receptor interaction and chemokine signaling. A recent genome-wide association study showed that the neuroactive ligand-receptor pathway was significantly related to risk of lung cancer [67]. Here, our interest is the chemokine signaling pathway, which is guided by interactions between *GNG11* and chemokines, such as *CXCL3*, *PPBP*, *CX3CL1*, *CXCR2*, and *CXCL13* (Figure 6c). Except for *PPBP*, none of these genes were drug targets. Additionally, the low druggability values for *GNG11* show that this gene cannot served as a potential target. As possible therapeutic alternatives, we suggest that key PPIs between *GNG11* and chemokines are not only key regulators of the chemokine signaling pathway, but also provide druggable possibilities due to their high TFC scores.

*GNG11* is a membrane-bound receptor that can be activated by chemokines via formation of a signaling complex [68]. To illustrate how the pathway is mediated by *GNG11*, a follow-up structural modeling of PPIs of *GNG11*-chemokines complexes was performed. Among the five predicted complexes, *GNG11* formed stable interactions with *PPBP*, *CXCL3*, and *CXCR2*, with binding energies of −19.87 kcal/mol, −128.16 kcal/mol, and −80.39 kcal/mol, respectively (Figure 6d). Structural modeling shows that *GNG11* binds to chemokine partners through the same or overlapping interfaces (Table S4), but adapted different conformations, either with long chain or curled forms. Accordingly, we provide a structural overview of *GNG11* signaling in terms of its competitive binding and consequences to other signaling pathways and regulatory process by forming transient interactions with chemokines. The dynamic shift of binding conformation may allow the intricacy of the cellular network and the heterogeneity of regulatory mechanisms [69].

Through these levels of analysis, we have demonstrated that module 2 provides a molecular space for interpreting chemical–protein interactions and drug target identification, suggesting that the chemokine signaling pathway and several *GNG11* involved interactions are potential therapeutic targets for NSCLC.

### 3.5. The Overall Network Reveals Critical Signaling Hubs and Regulatory Mechanisms

The overall community network consisted of two major communities (modules 1 and 2) and six small communities, including three connected and branched motifs that were also detected. GO analysis showed that these small motifs contributed to special biological functions. In addition to these isolated communities, two bottleneck genes (*ITGA1* and *MMP9*) were detected as a bridge that connected the entire community network. *MMP9* was the seed gene that connected module 1 and the *MMP* family-related motif. *MMPs* degrade various ECM components, destroy the histological barrier for tumor cell invasion, and play a key role in tumor invasion and metastasis [70]. As mentioned above, the *MMP* motif has the highest average TFC score, while *MMP7*-*MMP11* was the highest weighted edge among all small communities, suggesting it may act as a regulatory motif. Our result is agreement with a recent proteogenomics study of NSCLC, which showed that high *MMP11* and *MMP7* expression were significantly associated with poor overall survival [8]. In our community network, the *MMP* motif together with the adjacent motif drive ECM remodeling. These findings likely reflect modulation of the tumor microenvironment, with *MMPs* functioning as key players.

**Figure 7:**
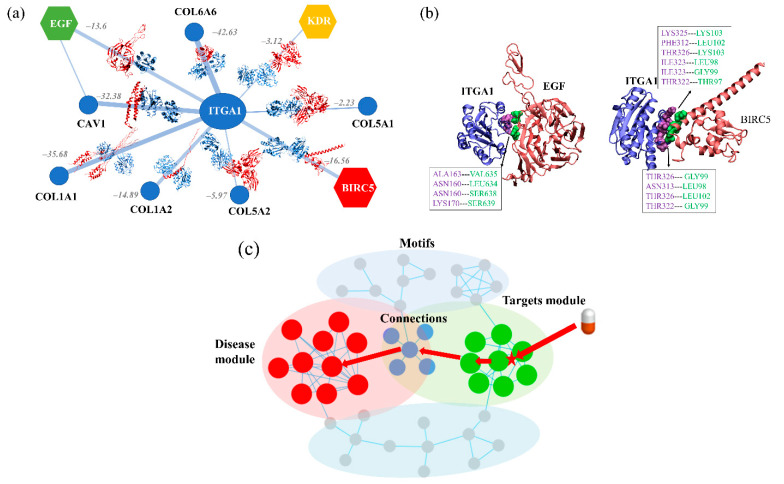
(**a**) The structural modeling of *ITGA1* and its interaction partners, while binding energy scores are also shown for edges. (**b**) The modeling structures of *ITGA1*–*EGF* and *ITGA*–*BIRC5* interactions, with their interacting residues are shown as surfaces. (**c**) The concept of a regulatory mechanism based on community network analysis. The target module (green circle) can regulate the disease-related module (red circle) through some connected motifs (overlapped circle) by targeting key protein–protein interactions (asterisk).

Most important was the bottleneck gene (Integrin alpha-1, *ITGA1*) between two of the major communities, which is a pre-malignant biomarker that promotes treatment resistance and metastasis potential in pancreatic cancer [71]. From the network perspective, *ITGA1* connects with three seed genes (*EGF*, *BIRC5*, and *KDR*) from different communities. In particular, the *ITGA1*–*BIRC5* interaction is the most important, as it was predicted with the highest TFC; the *ITGA1*-*EGF* and *ITGA1*–*KDR* interactions showed relatively high TFCs. By remodeling the *ITGA1*-related network by extracting all neighbors of *ITGA1* and their interactions from the WCN (Figure 2f)**, a new community that contained an additional six *ITGA1*–collagen interactions was obtained (Figure 7a). Similar to the *MMPs* motif, these *ITGA1*–collagen interactions may also involve tumor microenvironment and effect cellular behaviors and signal transduction pathways in NSCLC. Further structural modeling suggested that all interactions with *ITGA1* could be modeled, which distinguished two kinds of PPIs. One is that *ITGA1* forms two stable interactions with *BIRC5* in module 1 and *EGF* in module 2 that establish macromolecular complexes, connecting the two major communities. *ITGA1* uses different recognition regions when interacting with *EGF* and *BIRC5* (Figure 7b), generally binding with *EGF* by interfacial resides (such as ASN160, ALA163, and LYS170), while it binds *BIRC5* through another lager interfacial region. The other is that *ITGA1* forms transient interactions with several collagens. There is a competitive relationship between these transient interactions, with *ITGA1*-*COL6A6* being the most competitive (having the lowest binding energy: −42.63 kcal/mol), and they usually are involved in ECM-cell communication.

By investigating the role of *ITGA1* in connecting the two functional models, we proposed a regulatory mechanism based on the global architecture of the community network of NSCLC (Figure 7c). There are two fundamental assumptions that suggest the biological functions underlying the two major communities. Module 1 is related to some basic biological processes of carcinogenesis and NSCLC development, and thus can serve as the disease module. This module may play its biological functions by regulation of module 2. Activation of the chemokine signaling pathway starts by targeting several *GNG11*–chemokine interactions. Therefore, such module–module interactions may enable effective control of the regulatory mechanism via the high ‘control centrality’ of *ITGA1* and its bottleneck interactions.

## 4. Discussion

All cancers (including NSCLC) are caused by loss of mitotic control, which is governed by an intricate signaling network. Therefore, a systems-level understanding of the altered molecular mechanisms and cellular communications in cancer is still badly needed. In this study, we presented ne-PCA, an algorithm to build and analyze a “community network” based on high-throughput gene expression and PPI data from NSCLC. First, a seed-and-extend strategy was used to rank PPI network nodes as potential NSCLC-related genes. Second, a new edge-based score was introduced to measure the importance of PPI network edges that leveraged both topological information and GO knowledge. Third, we use GN algorithm and LPA-derived “community networks” to detect different functional modules and understand their underlying regulatory mechanisms. Compared with other cluster methods for PPI network analysis, there are two major advantages of our computational methods: (1) our approach adopted the complementary properties of two commonly used community detected methods, the GN algorithm, which use a “top-down” approach starting at the network level, and LPA uses a “bottom-up” approach starting at the protein level, to detect more robust communities. (2) in addition to community analysis, our computational framework included network prioritization for both nodes and edges in the PPI network. Node ranking was based on prior knowledge of the seed genes. For edge ranking, we defined a new measure, which not only considered network topology but also involved the intrinsic biology of protein pairs, to prioritize key regulators in NSCLC. Thus, our computational method is knowledge-guided and performed random work based on knowledge of seed genes and community detection based on GO similarity. The community network and underlying regulatory mechanisms provide a molecular understanding of how communications occur between different functional modules. Additionally, incorporating structural data to the community network gave the atomic insights into the signaling network and will help target PPIs for NSCLC therapy in the future [72].

After applying our method to two NSCLC expression microarray datasets, the final community network contained eight communities with different biological functions, revealing functional homogeneity of each community. Among them, the two largest communities, defined as module 1 and module 2, were found to control two distinct aspects of NSCLC. Module 1 contains 44 nodes, with 43 up-regulated genes involved in cell proliferation and NSCLC tumorigenesis. According to RWR scores, several important genes were predicted, including *CCNB1*, which had the highest RWR score. Cyclin B1 (*CCNB1*) binds to specific cyclin-dependent kinases, and these interactions play crucial roles in the cell cycle regulation [73]. It is well known that *CCNB1* is highly expressed in NSCLC and is a potential biomarker for both lung adenocarcinoma [74] and lung squamous cell carcinoma [75], belonging to two subtypes of NSCLC. Constructing the *CCNB1*-related subnetwork revealed that *PRC1*, *CEP55*, *KIF14*, *KIF4A*, and *HJURP* had the highest RWR scores. Alongside *CCNB1*, this sub-network presents a list of additional candidate genes with a strong survival association in NSCLC. We suggest that module 1 could serve as a disease module, as it is functionally enriched in the basic tumorigenic processes and could distinguish between lung tumors and normal samples with higher accuracy than the seed genes.

Compared with module 1, module 2 contained more down-regulated nodes, but most of the genes are known drug targets, highlighting the importance of this module for therapeutic opportunities. We have concluded that chemokine signaling pathway and *GNG11*–chemokine interactions may provide more promising drug targets for NSCLC from three levels of analysis: (1) the functional enrichment analysis showed that module 2 was functionally enriched for tumor-related signal transduction pathways, especially chemokine signaling. This pathway is of particular important and needs further investigation, because it has been reported as a biomarker for lung cancer [76]. (2) By using TFC scores to predict key PPIs in module 2, we have also found that *GNG11*–chemokine interactions are key regulators of the dysregulation of this pathway. Membrane-bound forms of chemokines allow communication with their receptors through direct cell-cell contact, which influences multiple fundamental biological processes and disease conditions, including cancer [77]. C-X-C chemokine receptor 2 (*CXCR2*) is a key chemokine receptors that has been shown to promote NSCLC cell proliferation, invasion, and stemness while suppressing apoptosis and chemosensitivity, via activating JAK2/STAT3 signaling [78]. (3) The structural modeling showed that both *GNG11* and chemokines in chemokine signaling pathway are not druggable, but *GNG11* could form stable physical interactions with *CXCR2* (DS = 0.834), *CXCL3* (DS = 0.721), and *PPBP* (DS = 0.85) by a series of interfacial residues, which may offer more promising hotspots for drug targeting.

The community network for NSCLC not only included functional modules as isolated entities that were responsible for specific cellular processes, but also included two connection nodes (*MMP9* and *ITGA1*), which are involved in processes that influence other nodes to accomplish higher-level cellular functions. Although they regulate different processes, *MMP9* and *ITGA1* are ECM regulatory proteins that directly participates in ECM assembly and turnover. Other metalloproteinases including *MMP1* and *MMP7*, have been proposed as prognostic biomarkers for NSCLC because high circulating levels of both proteins are associated with poor prognosis in NSCLC patients [79]. *ITGA1* is a typical adhesion molecule in cancer cells that mediates cancer cell behaviors, especially when combined with collagen [80]. Here, we emphasize novel functions, determinants of context dependency, and mechanistic-based therapeutic opportunities related to *ITGA1*. We suggest that the connected motifs include *ITGA1*- and *MMP*-mediated remodeling of the tumor microenvironment, which controls tumor development and metastasis [81]. Together, our findings suggest that targeting the ECM network, including *ITGA1*- and *MMP*-involved interactions, has potential therapeutic value.

It is widely accepted that cells are composed of different types of interacting modules, whose function is not played independently, but regulated with each other by both physical interactions and association networks. The landscape of the “community network” is based on global topology of the whole network, and intrinsic functions of each module define the mechanistic nature of the altered cellular communications in NSCLC. In line with our community network, we hypothesized two levels of communication: (1) intra-modular communication that is activated by targeting regulatory pathways and their key PPIs, which are the drug targets enriched in module 2; (2) inter-modular communications from module 2 that act as a signal transduction module to module 1 (the disease-related module) through small motifs. Our proposed regulatory mechanism is more or less similar to the allo-network model [82,83], which was proposed to study protein allosteric communication transmitted by PPIs within the cell. However, the community network based on PPIs only provides a simple framework for studying regulatory mechanisms; more regulators and their detailed molecular mechanism are needed to be considered, such as how miRNAs regulate PPIs, leading to tumor invasion and metastasis [84,85].

## 5. Conclusions

We have presented ne-PCA, an algorithm that identifies functional modules based on gene expression and PPI data. By applying ne-PCA to NSCLC, we generated a “community network” that was used to understand the molecular mechanisms of cancer. The “community network” identified a *CCNB1*-mediated network in the largest community as a modular biomarker, and interactions between *GNG11* and *CXCR2*, *CXCL3*, and *PPBP* in the second community provide potential druggable targets. Further structural modeling of PPIs in module 2 and the connected motif gives the complete in-depth functional landscape of NSCLC. We hope that this study provided insights into the molecular mechanism and biological functions that are altered in complex diseases both at the systems and molecular levels.

## Figures and Tables

**Table 1:** Information of expression microarray datasets.

Accessions	Platform	Samples (Cancer vs. Normal)
GSE19804	Affymetrix GPL570	60 vs. 60
GSE101929	Affymetrix GPL570	32 vs. 34
TCGA	Illumina HiSeq 2000	67 vs. 59

**Table 2:** Collection of known NSCLC genes (seeds).

Database	Inclusion Criteria	Gene Counts	URL
CGC	only genes’ tumour types as NSCLC were considered	42	https://cancer.sanger.ac.uk/census (January 2021)
KEGG	genes in PATHWAY: map05223 were collected	81	https://www.kegg.jp/kegg/ (January 2021)
DisGeNET	only genes’ diseaseName as NSCLC in curated gene-disease associations were considered	158	https://www.disgenet.org/ (January 2021)

## Data Availability

No new data were created or analyzed in this study. Data sharing is not applicable to this article.

## Supplementary Materials

The following are available online at https://www.mdpi.com/2073-4409/10/2/402/s1, Figure S1: Distributions of degree for primary (a) PPI and (b) WCN; Figure S2: KEGG pathway enrichment analysis of the primary PPI network; Figure S3: Four commonly topological parameter of genes in primary PPI network; Figure S4: Distributions of (a) edge betweeness and (b) TFC for edges in primary PPI network; Figure S5: Communities identified by GN and LPA models; Figure S6: Comparison results of first and second modules between ne-PCA and four classical topology module detection algorithms; Figure S7: KEGG pathway enrichment analysis of module 1; Figure S8: Remaining survival curves of prognostic genes in module 1. The higher the expression of all genes, the worse the prognosis; Figure S9: KEGG pathway enrichment analysis of module 2; Table S1: Commonly topological properties of primary PPI network; Table S2: Gene list of M1 and M2 detected by ne-PCA, MCODE, MCL, CLusterOne, and Moduland; Table S3: Drug target and Drug information for proteins in module 2; Table S4: Interacting residues for *GNG11-CXCR2*, *GNG11-CXCL3*, and *GNG11-PPBP* interactions.
