# Supplementary material for: Knowledge-Guided “Community Network” Analysis Reveals the Functional Modules and Candidate Targets in Non-Small-Cell Lung Cancer
Source: Cells. 2021 Feb 16;10(2):402. doi: 10.3390/cells10020402 (PMC7919838; doi:10.3390/cells10020402)
Supplement: Supplementary file 1 [file cells-10-00402-s001.zip › cells-1091849 - Supplementary Figures.pdf]

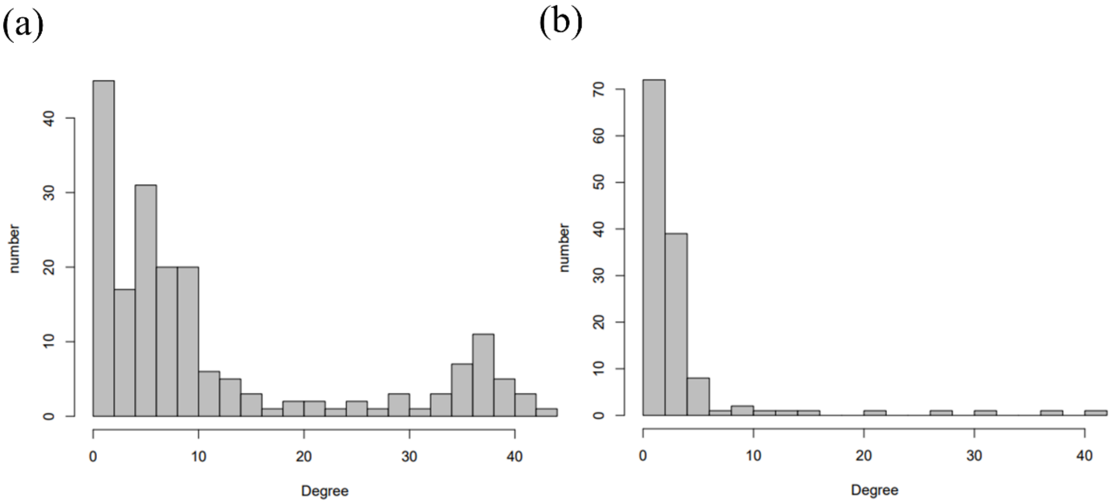

Figure S1. Distributions of degree for primary (a) PPI and (b) WCN.

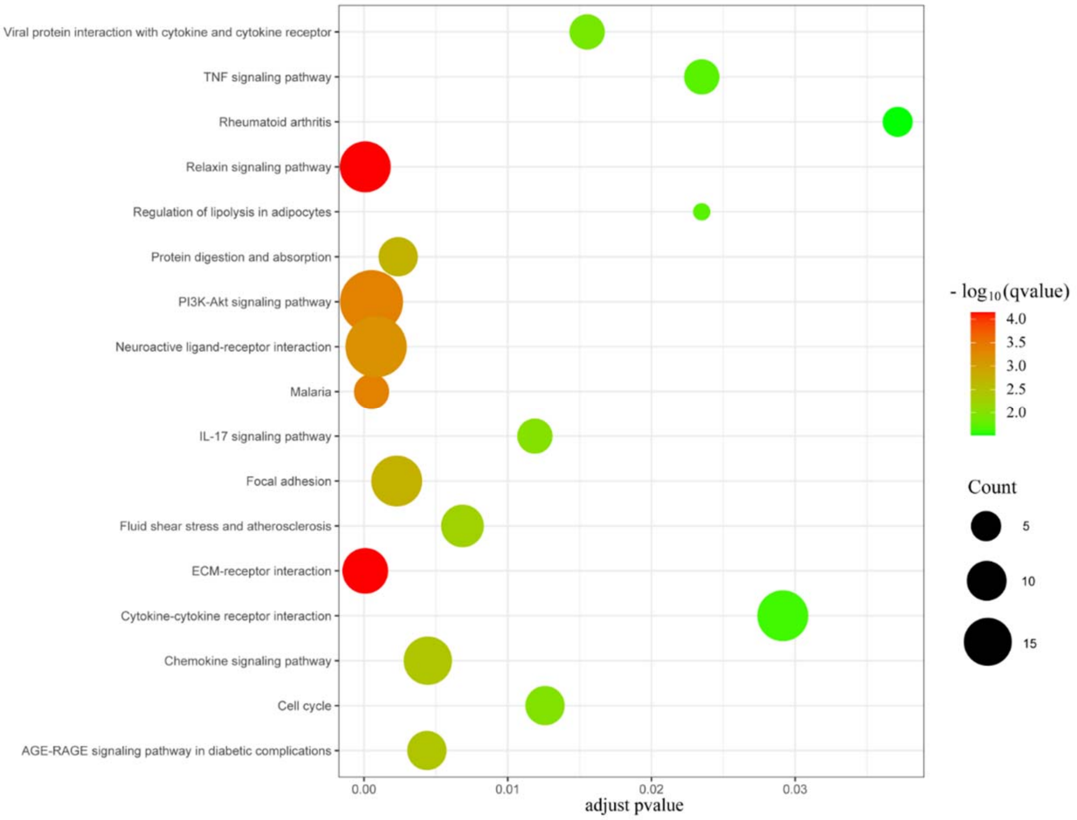

Figure S2. KEGG pathway enrichment analysis of the primary PPI network. The size of the dots represents the fraction of genes in the category that are expressed in the corresponding state, whereas the color indicates significance from green (less significant) to red (most significant).

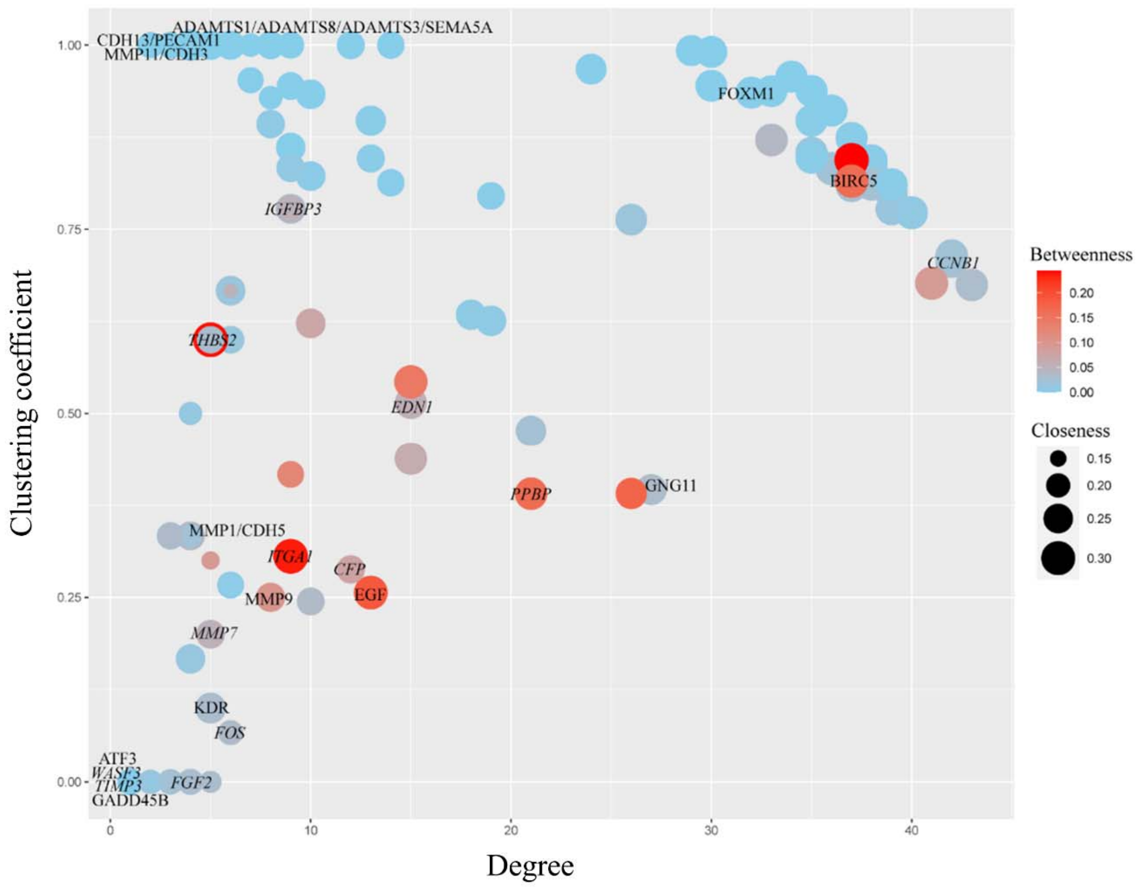

Figure S3. Four commonly topological parameter of genes in primary PPI network. The x axis and y axis represent the distribution of degree and clustering coefficient, respectively. In addition, closeness is represented by node size, and betweeness is represented by the color from red (large) to blue (small).

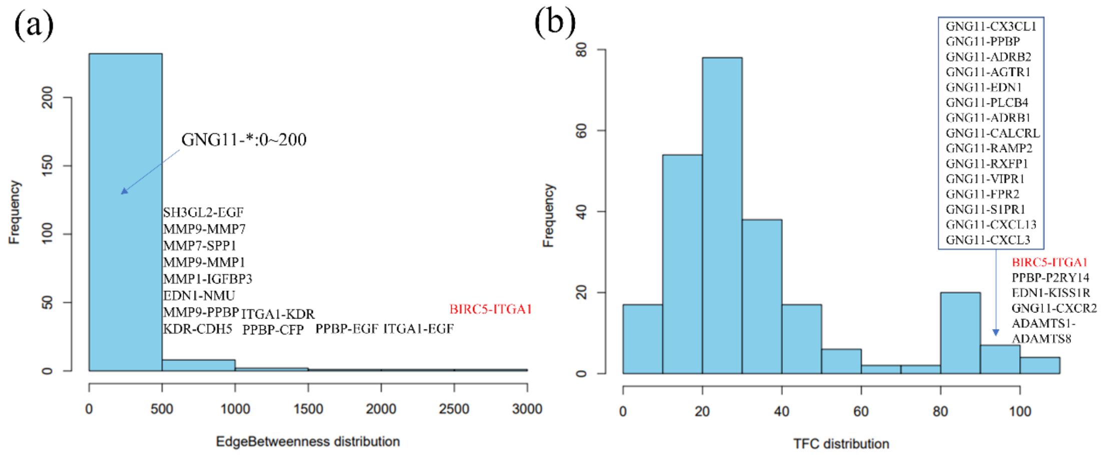

Figure S4. Distributions of (a) edge betweeness and (b) TFC for edges in primary PPI network.

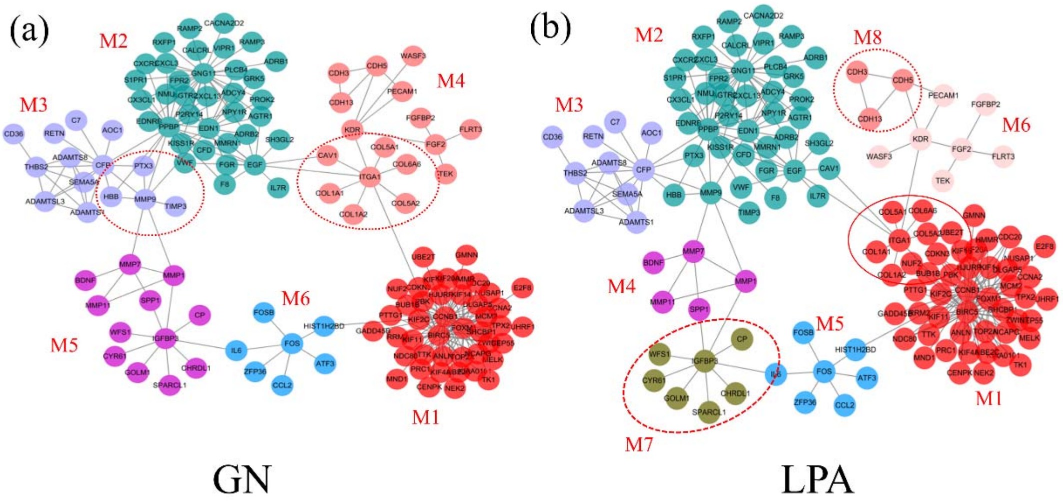

Figure S5. Communities identified by GN and LPA models. (a) Six communities in GN model. (b) Eight communities in LPA model. Different communities corresponding to Figure 3(a) were distinguished by different colors, and the topological difference between GN and LPA results were highlighted by red circles.

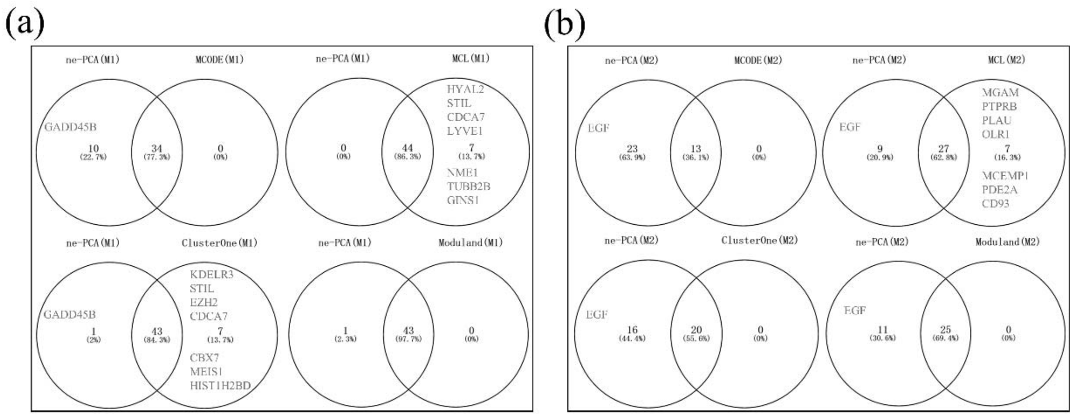

Figure S6. Comparison results of first and second modules between ne-PCA and four classical topology module detection algorithms.

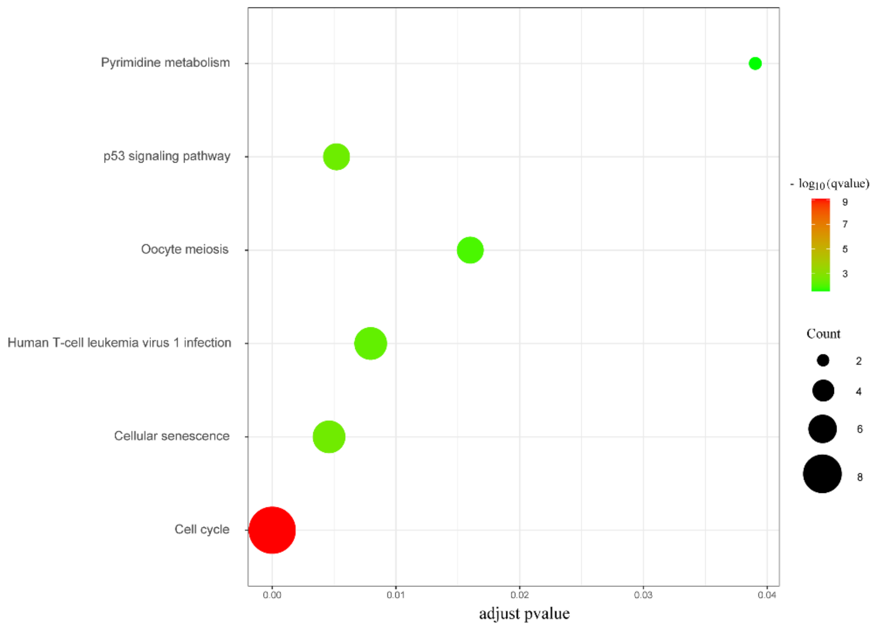

Figure S7. KEGG pathway enrichment analysis of module 1.

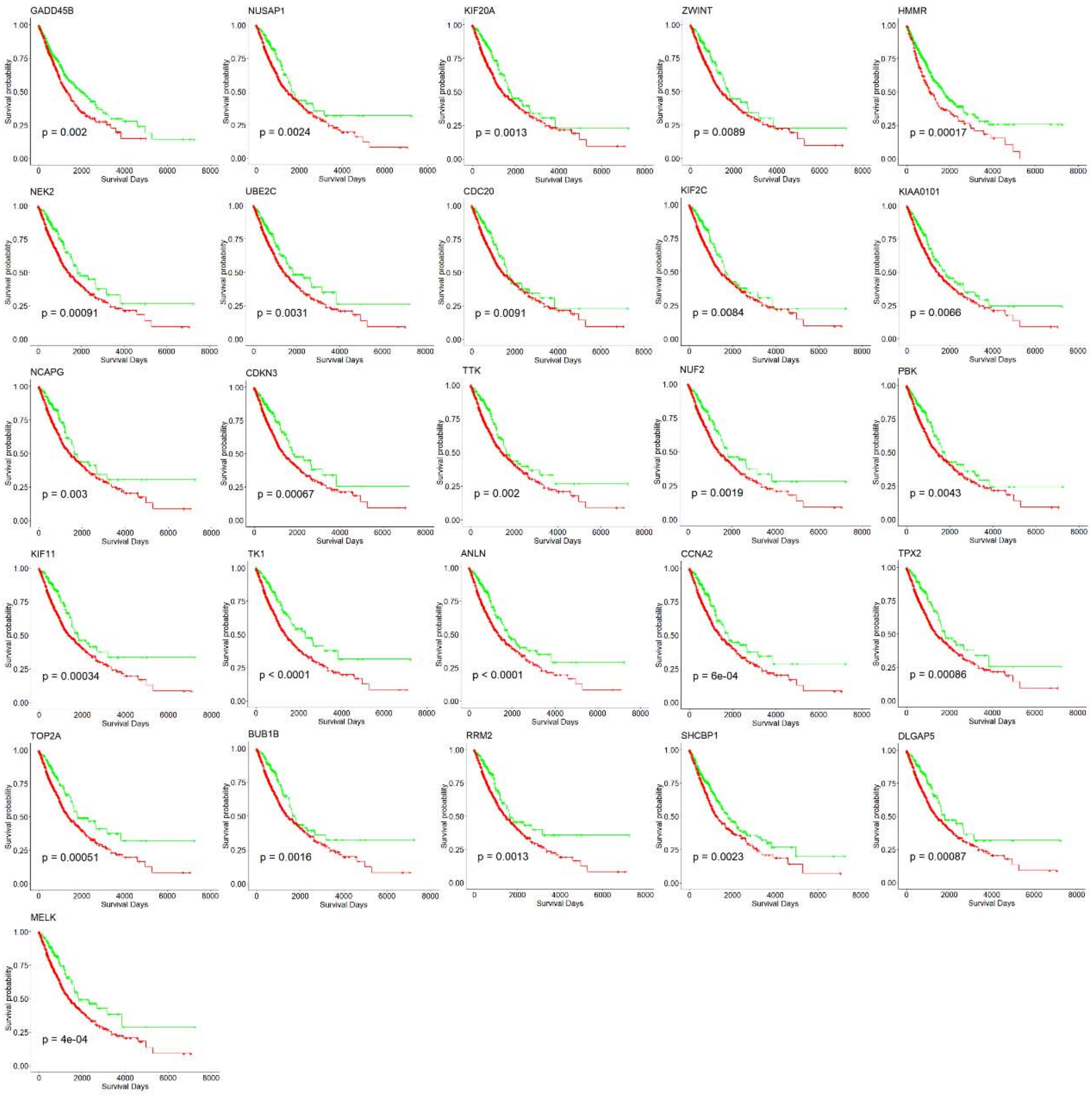

Figure S8. Remaining survival curves of prognostic genes in module 1. The higher the expression of all genes, the worse the prognosis.

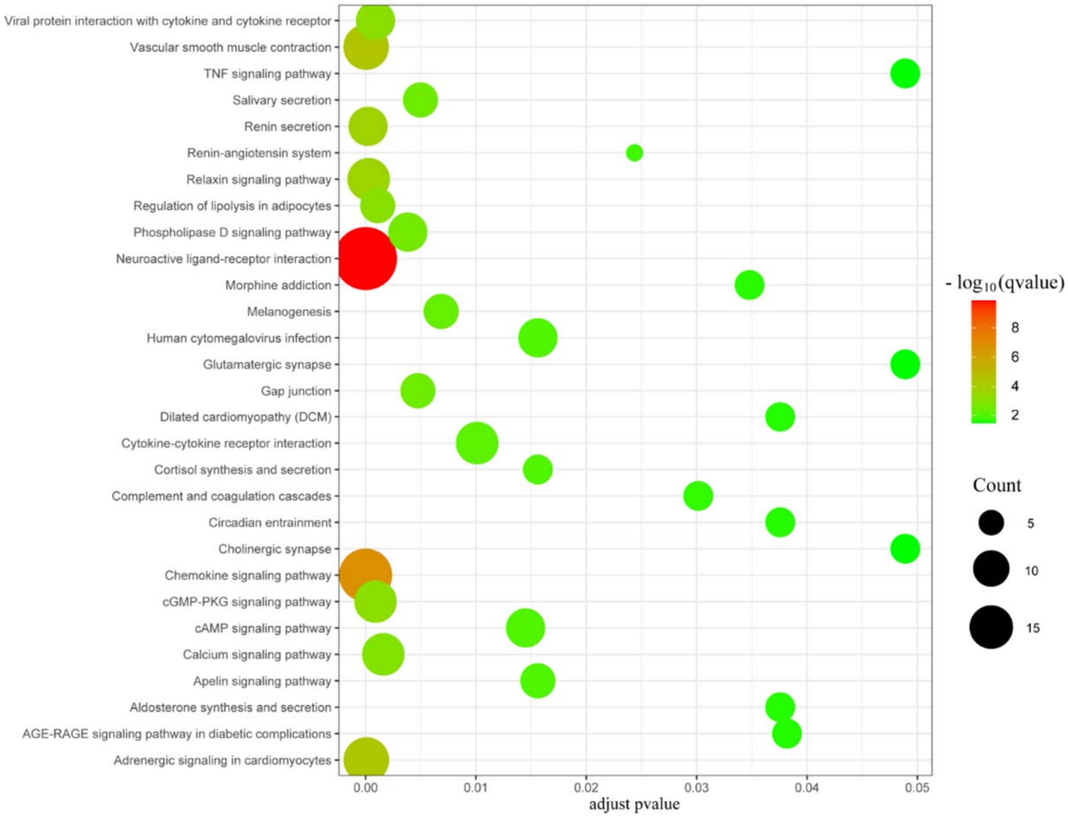

Figure S9. KEGG pathway enrichment analysis of module 2.
